# Beyond autonomy: unpacking self-regulated and self-directed learning through the lens of learner agency- a scoping review

**DOI:** 10.1186/s12909-024-06476-x

**Published:** 2024-12-23

**Authors:** Nidhi Gupta, Kamran Ali, Dan Jiang, Trine Fink, Xiangyun Du

**Affiliations:** 1 College of Dental Medicine, QU Health, Qatar University, Doha, Qatar; 2 Aalborg UNESCO PBL Centre, Department of Sustainability and Planning, Aalborg University, Aalborg, Denmark; 3 Aalborg UNESCO Center for Problem-Based Learning, Department of Sustainability and Planning, Aalborg University, Aalborg, Denmark; 4 Department of Health Science and Technology, Aalborg University, Aalborg, Denmark

**Keywords:** Learner agency, Self-directed learning, Self-regulated learning, Pre-doctoral dental education, Undergraduate dental education

## Abstract

**Background:**

Learner agency involves students actively engaging in their learning process and shaping their educational experiences through autonomy, self-regulation, and decision-making. In professional education, particularly within health professions, learner agency is critical for fostering adaptability and lifelong learning. This scoping review explores how learner agency, alongside concepts such as self-regulated learning and self-directed learning, is addressed in undergraduate dental education, aiming to understand its implications and strategies for enhancing student agency in this context.

**Methods:**

The scoping review examined literature on self-regulated learning, self-directed learning and learner agency in undergraduate dental education from 1994-April 2024 across five databases: PubMed, Scopus, Embase, ProQuest Central, and Web of Science. A manual search of the cited references was also conducted. Relevant studies were screened, and the findings were summarized to offer a comprehensive overview and identify research gaps.

**Results:**

In total, 33 studies were included in the review. The results revealed a strong interconnection between intrapersonal, behavioral, and contextual dimensions in shaping learner agency through self-regulated learning and self-directed learning among undergraduate dental students. The studies analyzed, predominantly quantitative, highlighted the multifaceted relationships among self-regulated learning and self-directed learning and learner agency, emphasizing its significance for educational practice and policy.

**Conclusions:**

Self-regulated learning and self-directed learning are crucial for developing learner agency, aiding undergraduate students’ transition into independent professionals and fostering lifelong learning behaviors. Educational strategies should prioritize empowering students to become independent learners, reducing their reliance on faculty. Further research is needed to identify effective methods for promoting learner agency development among dental students.

**Supplementary Information:**

The online version contains supplementary material available at 10.1186/s12909-024-06476-x.

## Introduction

Learner agency, a concept rooted in individuals’ active engagement in their learning process, refers to students’ will and capacity to act. It emerges from the dynamic interactions between students’ independent engagement in learning within specific sociocultural settings and the contextual elements that either facilitate or hinder their ability to act [1]. As professional programs in higher education increasingly prioritize decision-making, problem-solving, creativity, collaboration and navigating uncertainty, learner agency is gaining recognition [2, 3].This heightened attention stems from its relevance to student-centered approaches, academic performance and collaborative learning [3–5]. Considering the approaches to health professions education, learner agency (LA) is crucial, enabling individuals to control their learning process and adapt to the changing global health landscape. However, enhancing learner agency necessitates close attention to learner support mechanisms [6].

**Fig. 1:**
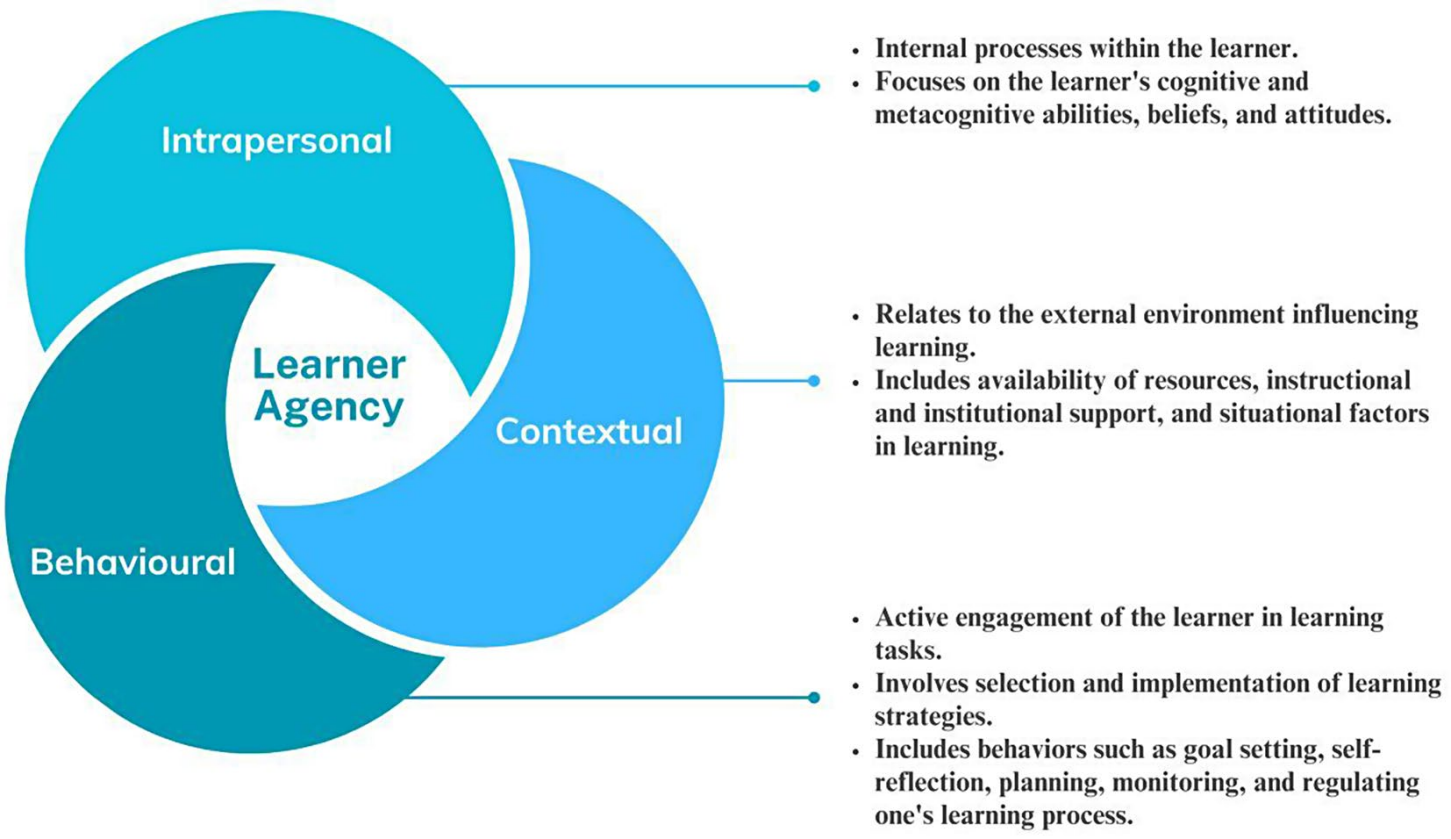
A framework for describing the dimensions of learner agency (*derived from Bandura*,* 2006*,* 2008* [3, 11]; *Du* et al., *2022* [10]; *Jääskelä et al.*,* 2017* [2]; *Mercer*,* 2011*,* 2012* [1, 7]; *Jiang* et al., *2023* [12]

LA draws from constructivist perspectives emphasizing individuals’ autonomy, self-regulation, and capacity to shape their learning experiences highlighting learners as active constructors of knowledge within their social and cultural contexts [1, 7]. According to Bandura’s concept of agency, agency functions within an interdependent framework known as “triadic reciprocal causation.” In this model, internal personal factors (such as cognitive, emotional, and biological events), behavior, and environmental factors act as interacting determinants that influence one another in a bidirectional manner [8, 9].Within this framework, learners are seen as agents who actively engage in their learning journey, make choices, set goals, and monitor their progress. Based on this, the current literature on LA suggests an integrated three-dimensional framework to conceptualize it [3, 10, 11].This framework, as described in Fig. 1, delineates LA across three interconnected dimensions:1) intrapersonal, 2) action (behavioral), and 3) contextual (environmental).

Several concepts closely related to learner agency include self-direction, decision-making, problem-solving, creativity, collaboration, self-regulation, and the ability to handle uncertainty. Owing to its focus on these areas, learner agency is gaining increasing attention in professional programs within higher education [4, 13, 14]. Studies have shown that self-regulated learning (SRL) is closely aligned with the concept of agency [13–16]. Defined as the process where one is ‘metacognitively, motivationally, and behaviorally proactive in the learning process’ [17], SRL has garnered increasing recognition as an important attribute in contemporary health professions education (HPE) [18, 19]. In educational contexts, self-regulation entails conscious awareness and the deliberate selection and application of suitable strategies to achieve learning objectives, whether explicit or implicit [18, 19]. As learners navigate their educational journey, agency evolves through ongoing reflection and evaluation of task progression [14]. When students perceive themselves as agents in their learning process, they are more likely to utilize self-regulatory strategies effectively [16]. This symbiotic relationship between agency and self-regulated learning underscores the importance of empowering learners to take ownership of their learning experience.

Self-directed learning (SDL) is another concept that is also addressed in the HPE and dental education literature [20–22]. Knowles (1975) defined SDL as a process whereby individuals take the initiative, with or without the help of others, to diagnose their learning needs, establish learning objectives, identify the necessary human and material resources for learning, select and implement suitable learning strategies, and assess the outcomes of their learning efforts [23]. SDL is being used in medical education, and it has been noted that medical students are playing an increasingly significant role in shaping their education and determining the measures needed to ensure that the growth of SDL aligns with the educational goals of the medical field [24]. For medical students to become self-directed learners, the competencies they need to acquire include the ability to identify their own learning gaps in skills and set goals for learning. Additionally, they must develop self-awareness, evaluate human and material resources for learning, engage in critical thinking and reflection, perform critical appraisals, and manage information effectively. Teamwork, self-evaluation, and peer evaluation are also essential skills that contribute to their growth as self-directed learners [25, 26].

SDL and SRL, though distinct in many ways, are closely related concepts and often used interchangeably in healthcare education literature. Therefore, it is important to see how they translate into LA which is the goal of transformation of healthcare graduates into independent professionals. Both approaches can be viewed through dual dimensions: external processes or events and internal factors such as personality traits and aptitudes. In practical terms, they encompass four key phases: defining tasks, setting goals and planning, enacting strategies, and monitoring progress while reflecting on outcomes [15, 23, 27, 28]. Importantly, intrinsic motivation serves as a driving force in both approaches, emphasizing the internal desire and enthusiasm that learners cultivate to pursue their educational goals autonomously. These shared characteristics highlight how SDL and SRL empower learners to take charge of their learning, promoting deeper understanding and long-term retention of knowledge [27].

The active engagement of the learner, along with making choices and decisions regarding learning strategies and reliance on metacognitive and cognitive operations such as self-efficacy and self-awareness, are key aspects of both SRL and SDL [29]. Nevertheless, it is important to recognize that SRL commonly occurs within classroom settings, is rooted in cognitive psychology, and emphasizes the learning processes associated with a task. In SRL, tasks are usually set by teachers, who focus on the internal cognitive and metacognitive processes that students use to manage their learning. This makes SRL a narrower, microlevel construct that is closely tied to formal educational settings [30]. On the other hand, SDL involves learners designing their learning environments and planning their learning trajectories, making it a broader, macrolevel construct that emphasizes autonomy and lifelong learning [29]. Moreover, it takes place in diverse environments and often involves self-regulated learning [28].

Recent studies give more weight to a complexity lens to conceptualize agency [4]. The complexity theory of change and development is frequently utilized in educational contexts to emphasize the need for self-organizing, dynamic education systems that can effectively respond to evolving societal shifts [31]. Complexity theory enables us to connect the elements of education to the three dimensions of learner agency identified by Bandura [11]. Morrison posits that this approach recognizes learning as a nonlinear and intricate process rather than a straightforward sequence and underscores the inseparable integration of the numerous factors influencing learning [31]. Such a conceptual shift supports the arguments for the literature to move from SRL and SDL to LA [4].

To comprehensively address the complexity of LA within undergraduate dental education, this paper provides a scoping review of how LA has been conceptualized and applied. The review undertakes a focused examination of associated concepts such as SRL, SDL and LA. Its primary objective is to gain a comprehensive understanding of LA and its ramifications in dental education. Guided by the research question “How is the learner agency of dental students addressed in contemporary undergraduate dental education programs?”, this review seeks to elucidate the strategies and approaches employed in addressing learner agency within current undergraduate dental education programs.

## Methods

A scoping review was opted for instead of a systematic review, as the aim of this study was to identify knowledge gaps, map out a body of literature, clarify concepts, or explore research methodologies [32, 33].Unlike systematic reviews, which typically synthesize existing evidence on relationships between exposure and outcome variables, scoping reviews are designed to map the breadth and depth of research activity on complex topics and identify gaps in the relevant literature [34]. In this scoping review, we employed the five-stage framework proposed by Arksey and O’Malley [34], which includes (1) identifying the research questions, (2) identifying relevant studies, (3) selecting the relevant studies, (4) charting the data, and (5) collating, summarizing, and reporting the results. Checklist to demonstrate compliance with Arksey and O’Malley framework is attached as Appendix 1. This approach enabled our study to make significant contributions by providing a comprehensive and explicit summary of the available evidence on LA, SRL and SDL among undergraduate dental students.

### Step 1: research question and protocol registration

The purpose of our review was to investigate how LA is addressed through SRL and SDL in undergraduate dental education guided by the following research question:

- What are the ways in which the learner agency of dental students is addressed through SRL and SDL in current undergraduate dental education programs? (intrapersonal, behavioral, environmental)

The manuscript followed the Preferred Reporting Items for Systematic Reviews and Meta-Analyses Extension for Scoping Reviews (PRISMA-ScR) guidelines [32], with the PRISMA-ScR checklist attached as Appendix 2. The protocol was registered on the Open Science Framework (OSF) platform [35].

### Step 2: identifying relevant studies

#### Eligibility criteria

The preset inclusion and exclusion criteria were well defined, ensuring that the literature search targeted studies specifically related to LA, SRL and SDL within the undergraduate dental curriculum.

#### Inclusion criteria

- Primary research studies on SRL and SDL published in the last 30 years, i.e., January 1994 to April 2024.
- Full-text studies published in English.
- Population: Undergraduate dental education.
- Types of manuscripts: peer-reviewed journal articles and conference papers.

#### Exclusion criteria

- Date: Prior to Jan. 1994 and after April 2004.
- Language: Not written in English.
- Postgraduate dental education and other disciplines within medical education, K-12 education, and vocational education.
- Types of manuscripts: Conference abstracts, seminars, opinion papers such as editorials, commentaries, reviews of literature, grey literature, book chapters or articles not meeting the inclusion criteria.

#### Information sources and search strategy

A comprehensive search of electronic databases was conducted up to the last 30 years (January 1994–April 2024). The literature from five relevant databases, namely, PubMed, Scopus, Embase, ProQuest Central and Web of Science, was included in the review. Additionally, as suggested by Booth et al., a manual search of the references cited by the included studies and their respective references was performed [36].

The search strategy was developed by combining Medical Subject Headings (MeSH) and keywords specific to PubMed, along with index terms relevant to other databases (Table 1). Boolean operators, truncation, and phrase searching were integrated into the search strings to ensure the creation of meaningful and comprehensive search queries. This process involved consultation with an experienced librarian to optimize the search strategy.

**Table 1:** Search strategy used to explore electronic databases

Block 1	self-regulate* OR self-organize* OR self-direct*OR self-orient* OR self-led OR self-regulated learning OR “self-directed learning”
Block 2	undergrad* OR undergraduate dental education
Block 3	dental students OR dentist

### Step 3: selecting the relevant studies

All identified articles were imported into reference management software EndNote^®^ X9 (Clarivate Analytics, London, UK). After removing duplicate articles, two authors in this study (NG and DJ) independently screened the remaining articles based on their titles and abstracts via Rayyan Systematic Review Screening Software [37]. Disagreements were resolved through discussions between the two authors (NG and DJ), and the third author helped to moderate any residual differences through collaborative discussions aimed at reaching consensus. Articles meeting the eligibility criteria after full text review were included in the scoping review.

#### Study selection

**Fig. 2:**
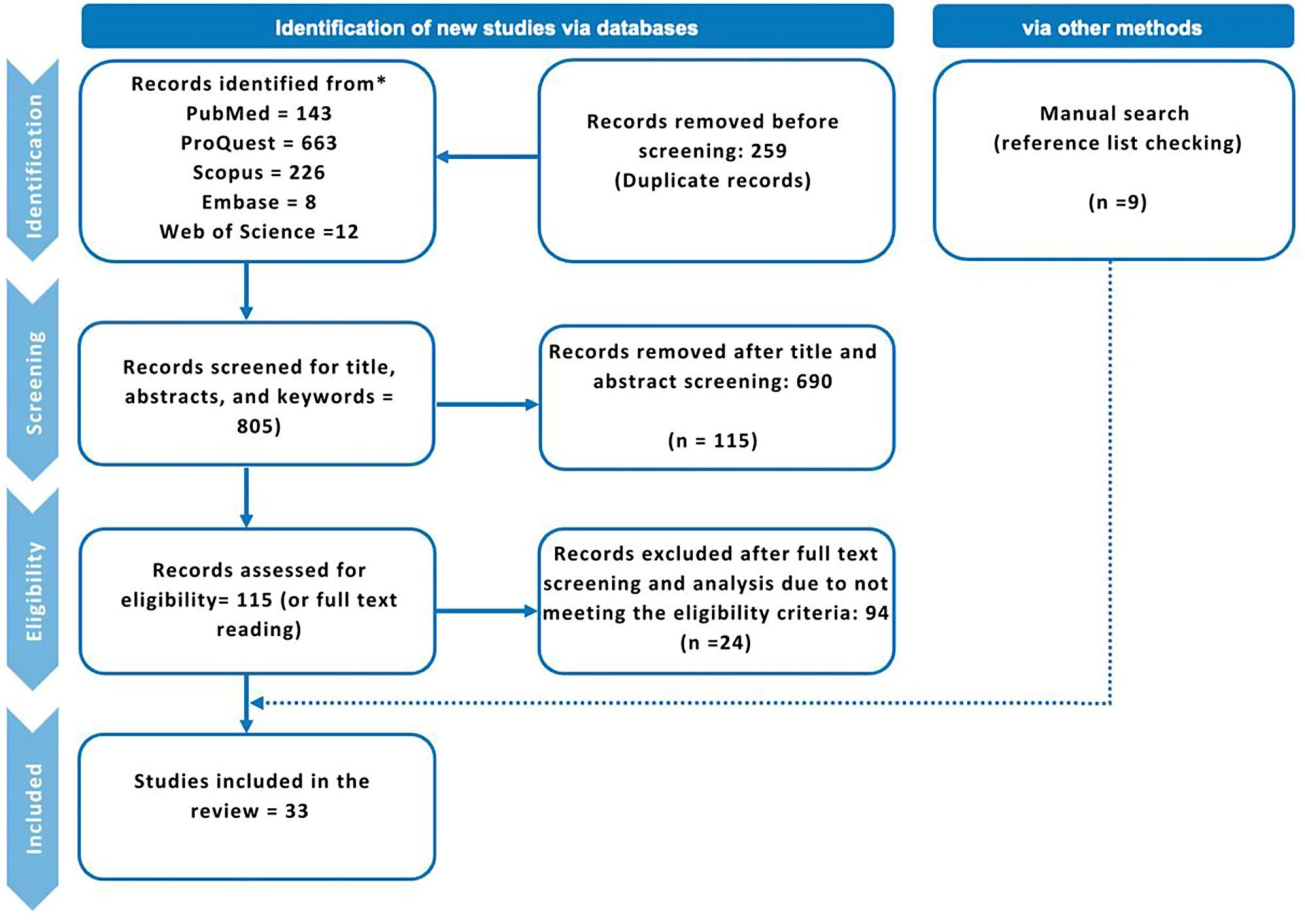
PRISMA Flowchart of Search Results

The results of the literature search and study selection are depicted in the flowchart (Fig. 2). The selection process followed the methods of identification, screening, eligibility, and inclusion as described by Liberati et al. [38]. Initially, the review focused on titles and keywords or topics retrieved from the five databases to keep the results manageable. The initial search yielded 1,064 articles. After 259 duplicates were eliminated, 805 articles were further screened. The following criteria were used for the review and selection of studies: availability in English and focus on SRL, SDL, and LA in undergraduate dental education. Articles that reported undergraduate dental student data when students from other programs (e.g., medical students, nursing, pharmacy, postgraduate students) were also involved in the study were included [39–45]. Other scoping and systematic review papers on SRL and SDL in medical education were not further analyzed because of their limited information on dental education. Therefore, these literature review papers served solely as tools for reference list checking. The first author (NG) screened the titles and abstracts for relevance to the research questions, leading to the exclusion of 690 articles that did not meet the inclusion criteria. This included studies with irrelevant titles, keywords, and abstracts from other health professional fields not related to dental education, as well as systematic reviews and conceptual papers. The selection process was conducted twice to ensure that no relevant publications were mistakenly excluded. A full-text screening was subsequently conducted for 115 articles, and a total of 45 studies were selected for further open-coding analysis. This was followed by a full-text analysis in which another 21 articles were removed specifically for not identifying the components of LA. Given that electronic searches may miss significant published studies due to indexing limitations, errors, inaccuracies, or concepts lacking appropriate subject headings, we supplemented our search by manually [36] examining the reference lists of the remaining 24 studies and relevant systematic/scoping reviews, and an additional 9 relevant journal articles were added. Finally, 33 articles were included in the qualitative analysis. Consensus was maintained throughout the article selection process, ensuring consistency in the identification of LA themes. A codebook encompassing LA themes and references was established, and participant information was extracted by the first author.

### Step 4: data charting and coding process

Data charting was carried out via Microsoft Excel, and a codebook was developed to ensure consistency in data extraction. This enabled the capture of essential data items, as outlined in Table 2. The data items extracted during charting included the author’s name, year of publication, source, type, country, research method, theories and analytical framework, participants, context, and LA themes. The extraction was performed by the first author, NG.

The auditing process involved a second reviewer (DJ), applying a thematic approach combined with open coding and referring to the provided codebook for extraction. This step was undertaken to ensure validity. Interrater reliability (IRR) was also assessed on a subset of 10% of the studies in this phase, with the IRR results showing an acceptance rate of over 0.85 for each theme. Each coder independently extracted data to a codebook using the same criteria. The second coder was blinded to the data provided by the first author. The results were based on LA themes extracted from the data. The characteristics of the included studies are as follows:

#### Sources of publication

The review encompassed various journals, with the European Journal of Dental Education being the most frequently cited, contributing eight articles. The Journal of Dental Education followed with five articles, whereas BMC Medical Education provided three. Other journals included the Journal of Clinical and Experimental Dentistry, the Journal of Education and Health Promotion, and Acta Odontológica Latinoamericana, each with two articles. Several journals were represented by a single article each: Medical Education, Pakistan Orthodontic Journal, Canadian Medical Education Journal, African Journal of Health Professions Education, Professional Medical Journal, Journal of Medical Education and Curricular Development, Academic Bulletin of Mental Health, Journal of Medical Internet Research, Tidsskriftet Læring og Medier and Frontiers in Psychology. This wide range of sources reflects the interdisciplinary interest and comprehensive examination of the topic across different fields and regions.

#### Country

Given the diverse educational contexts across various countries, contextual factors, such as the study setting, were considered crucial aspects in reporting the findings [46]. All the studies provided information on their respective study settings, which were distributed as follows: 9.3% in the U.S. and Korea; 12.5% in Pakistan; and 6.25% each in Australia, Syria, Chile, Qatar, and Saudi Arabia. The remaining studies were conducted in India, Belgium, Brazil, the UK, the Philippines, Denmark, Canada, Malaysia, Argentina, Turkey and Finland.

#### Year of publication

**Fig. 3:**
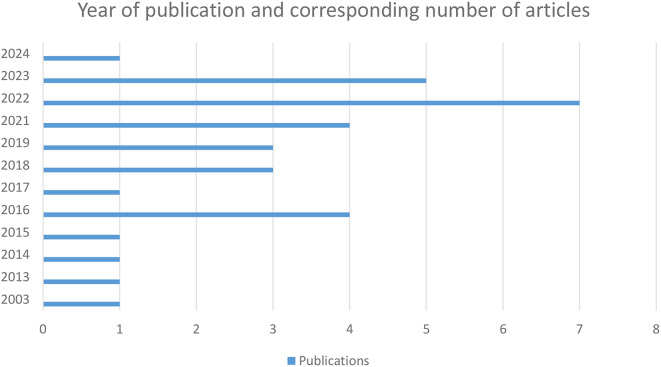
Year of publication of studies included in the review from 1994 to 2024

Although the search for articles spanned from 1994 to 2024 (Fig. 3), the first paper related to SRL/SDL/LA in undergraduate dental education appeared in 2003, indicating that these topics have gained popularity only in the last two decades. Interest in the subject grew significantly after 2016, reaching its peak in 2022.

#### Theories and analytical framework

The theories and analytical framework were specified in 26 out of 33 articles. Several studies have used multiple theories or models in their study design and analyses and can be categorized as follows: (1) active learning theories (e.g., problem-based learning (PBL), experiential learning) (*n* = 6); (2) theories related to SRL (*n* = 10); (3) SDL (*n* = 4); (4) theoretical models (e.g., theories on transitional psychology or organizational socialization theory, self-determination theory, assimilation theory of meaningful learning, sense of coherence) (*n* = 4); and (6) other theories (e.g., facets of reflective thinking, transaction distance theory) (*n* = 2). SRL was the most frequently employed, indicating that in discussions about learner agency, the behavioral dimensions in dental education are the most explored.

#### Research methods

Across the 33 studies investigating LA, SRL, and SDL, a wide array of research methodologies are apparent, showcasing diverse approaches to comprehending these concepts. Quantitative methods predominated, with 20 studies employing numerical data and statistical analysis to explore these educational phenomena. These studies used various approaches, including self-reported and self-evaluated questionnaires [42, 47–50], pre- and post-course questionnaires [21], and cross-sectional surveys/studies [44, 51–53]. Some studies have employed descriptive statistics [40, 54–57]. For example, Loka et al. used a reflection questionnaire to assess habitual action, understanding, reflection, and critical reflection on a 5-point Likert scale [49]. Orsini et al. conducted a longitudinal study collecting data on demographics and students’ motivation for attending university, measured through the 28-item Academic Motivation Scale [58]. Lan et al. utilized a massive open online course and quantitatively analyzed data via k-means clustering to identify five SRL behavioral indicators of student activity [43]. Notably, Alfakhry et al. conducted quantitative studies via a quasi-experimental study design [59, 60].

In contrast, six qualitative studies focused on in-depth exploration of SRL via focus group discussions and semi structured interviews [45, 61–65]. For example, in the qualitative study by Varthis et al., students responded to an online questionnaire followed by a group discussion aimed at problem solving before and after the blended learning experience [65].

Furthermore, mixed-methods studies, numbering six, combined qualitative approaches such as interviews and focus group discussions with quantitative approaches such as surveys, questionnaires, and pre- and post-tests [41, 66–70]. For example, Lee et al. [70] utilized qualitative analysis alongside a cross-sectional web-based survey, whereas Malau-Aduli et al. [66] employed quantitative analysis of survey data via descriptive statistics and thematic analysis guided by the conceptual framework of organizational socialization theory.

### Step 5: collating, summarizing, and reporting the results

The mapping results and information were gathered, summarized, and reported. Key themes were identified and consolidated to integrate and synthesize the literature, facilitating a clear and concise interpretation of the findings.

## Results

### Ways in which LA is addressed

This section presents the findings of the integrated data analysis. Building upon the conceptual framework outlined in the introduction, which delineates three themes from the literature, this analysis examines the characteristics of SRL and SDL in relation to the development of LA across these dimensions. The framework was initially used to guide deductive analysis, followed by inductive analysis. Table 2 provides an overview of the three dimensions, namely, Intrapersonal, Behavioral, and Contextual, which are further elaborated upon in the subsequent discussion.

**Table 2:** Themes of learner agency in self-regulated learning and self-directed learning

Themes	Subthemes	Frequency	Content
Intrapersonal	Self-efficacy	7	-Efficacy on internet and online learning, comparatively higher in females [47, 57] -Developing self-efficacy [5, 45, 52, 55, 70] -Varying self-efficacy levels across study years [47, 49]
	Motivation	10	-Students motivation for learning [41, 44, 48, 61–63, 65, 66] -Impact of clinical contact on motivation [58, 59, 64, 66]
	Knowledge acquisition	13	-Recognizing learning opportunities [40, 48, 49, 65–67] -Adopting different learning styles [42, 43, 48, 50, 51, 57, 61, 62]
	Skill development	4	-Transversal skills [21, 66] -Clinical reasoning skills [64] -Clinical application of skills [57, 60]
Behavioral	Learning Strategies	3	-Goal setting [41, 45, 59]
		3	-Planning [41, 45, 54, 56]
		3	-Monitoring [40, 41, 54, 59]
		11	-Self-reflection [45, 54, 59, 62, 64, 69] -Self-perception of academic performance [40, 44, 45, 49, 50, 52, 53, 55, 65]
		6	-Self-evaluation/Self-assessment [41, 48, 53, 59, 60, 69]
Contextual	With peers	5	-Peer support from students in other years [62, 70] -Active discussion and interactions with peers within the class [21, 45, 62, 66, 70]
	With supervisors	9	-Support from the supervisor [21, 42, 44, 45, 48, 51, 56, 62, 66, 70]
	With institutions	17	-PBL as an effective strategy to support student autonomy [5, 50, 61] -Support for various learning formats (flipped classroom, blended learning and so on) [21, 56, 65] -Institutional support on early and gradual clinical contact experiences [50, 57, 63, 64] -Experiential courses emphasizing patient‒physician/dentist communication [64, 69] -Cognizant learning and teaching environment [42, 55] -Access to resources and courseware provided by institutions [21, 48, 51, 53, 56, 57, 68, 70]

#### Intrapersonal

Self-efficacy, motivation, knowledge acquisition, and skill development are among the key subthemes associated with the intrapersonal dimension of LA, and they are significantly related to SRL and SDL. Studies have highlighted the critical role of self-efficacy in student success [21, 45, 47, 52, 57, 61, 64]. In online learning environments, self-efficacy is notably greater among female undergraduates and fourth-year students [47, 57]. However, Postma noted that some students may have lacked the self-efficacy beliefs necessary to meaningfully participate in case study exercises, indicating that self-efficacy is not uniformly distributed and may impact engagement and performance [64]. Motivation was a key theme across several studies [41, 48, 58, 61, 62, 65, 66], with clinical experiences having a particularly significant impact [58, 59, 64, 66]. Bowman noted that participants felt positive and inspired by their first-year dental surgery course, finding enjoyment in early clinical experiences and engaging in course content despite the challenges [62]. The theme of knowledge acquisition was prominent, with undergraduate dental students demonstrating effectiveness in acquiring subject-matter knowledge and showing increased enthusiasm for learning and embracing innovative educational approaches [5, 42, 43, 48–51, 56, 57, 62, 65–67].

#### Behavioral

In the realm of the behavioral dimension within LA, thematic analysis has identified several factors closely associated with both SRL and SDL. Key learning strategies include goal setting, identified in three studies [41, 45, 60], planning [41, 45], monitoring, noted in four studies [40, 41, 54, 59], and self-reflection, highlighted in six studies [45, 54, 59, 60, 62, 64].

These studies also delve into additional competencies crucial for professional development, including students’ perceptions of academic performance and their ability to self-evaluate [44, 45, 49, 50, 52, 53, 55, 65]. For example, Mehboob et al. reported that students actively establish their learning goals and are adept at selecting appropriate strategies to achieve them, scoring 3.81 on average for setting goals and 3.72 for planning and implementation [41]. High achievers tended to set both process-oriented and outcome-based goals rather than focusing solely on outcomes. They chose goals of medium difficulty that were achievable within a predetermined timeframe. By continuously self-evaluating and adjusting their learning behavior, high achievers monitor their progress and align their actions with their goals. They also possessed a reflective attitude, regularly reflecting on every step of their examination process [45]. In the study conducted by Malau-Aduli et al., the students acknowledged the importance of proactive planning for the day and their readiness to seek clarification on challenging topics [66]. They also realized the necessity of putting in more effort into developing problem-solving and self-directed learning strategies. Among the four aspects of the behavioral dimension, one-third of the studies have demonstrated a strong association between self-reflection and self-evaluation and improved self-regulated learning outcomes. Specifically, it is suggested that dental students who regularly engage in self-reflection and self-evaluation show better academic performance, develop critical thinking and problem-solving skills, and improve their overall learning. These practices may enable learners to become more effective, autonomous, and motivated, which in turn can translate into improved performance and academic success.

LA may not only encompass foundational learning strategies but also metacognitive skills that support students in navigating their educational journeys and preparing for future careers. These findings indicate the multifaceted nature of LA, hinting at its potential role in fostering adaptive and proactive learning behaviors among students.

#### Contextual (environmental)

The contextual dimension highlights the impact of external factors on an individual’s agency in learning. In this dimension of LA, thematic analysis identified critical areas that enhance SRL and SDL through interactions with peers, supervisors, and educational settings. Peer support plays a significant role, with studies emphasizing the benefits of active discussions and interactions among classmates, as well as support from students in other years [21, 45, 62, 66, 70]. Supervisory support is also crucial, with nine studies indicating that guidance and feedback from supervisors enhance student learning [21, 42, 44, 45, 48, 51, 62, 66, 70].

Institutional support is paramount and includes several effective strategies. A PBL environment promotes student autonomy [50, 61], whereas various learning formats, such as flipped classrooms and blended learning provided by institutions, cater to diverse preferences [21, 56, 65]. Early and gradual clinical contact experiences help students integrate theoretical knowledge with practical skills [50, 57, 58, 64], and experiential courses that emphasize patient‒physician‒dentist communication prepare students for real-world interactions [64, 69]. A conducive learning environment provided by institutions is crucial for student success [42, 55], and access to resources and courseware further supports students in their academic journeys [21, 48, 51, 53, 56, 57, 68, 70].

### Synthesis of results

The results suggest connections between intrapersonal, behavioral, and contextual dimensions in the interplay of SRL, SDL, and LA. These dimensions may collectively influence the development of LA among undergraduate dental students. Among the studies analyzed, eight used qualitative methods (25%), while quantitative methods were used more frequently (31.25%). These studies provide varied perspectives on how SRL and SDL might be linked with LA underscoring the complex relationship SRL and SDL with LA its possible implications for educational practice and policy.

## Discussion

To the best of the authors’ knowledge, this scoping review is the first to explore the interplay between SRL, SDL and LA in undergraduate dental education. These concepts have become a focal point in medical education primarily over the past two decades. Therefore, reviewing literature from the last 30 years was deemed both manageable and relevant, as few studies, if any, were conducted before the turn of the millennium. Literature published before 1994 is less likely to align with contemporary learning practices in medical education. Nevertheless, this review was extended to include works from 1994. By synthesizing the literature, it maps the current landscape of SRL and SDL implementation strategies, interventions, and assessment methods employed in undergraduate dental curricula, linking them to LA. The insights into the collective agency of these learners appear to be in line with Bandura’s claim that, by combining their shared knowledge, skills, and resources, individuals can collectively use their agency to shape their own environment [3]. To connect these concepts, this study proposed a framework for conceptualizing LA in relation to SRL and SDL, aiming to understand LA development in undergraduate dental education. The study results suggest that these three dimensions of LA (intrapersonal, behavioral, and environmental) are interactive and interrelated in the SRL and SDL literature, highlighting the importance of integrating these concepts to enhance the educational experiences and outcomes of dental students.

Research indicates that an internal locus of control, self-efficacy, and SDL are significantly linked to academic success [52]. Students who believe that they have control over their learning outcomes (internal locus of control) and possess confidence in their abilities (self-efficacy) are more likely to engage in SDL, leading to better academic performance. Another study revealed that self-directed learning readiness was positively correlated with academic performance [67]. These elements collectively enhance the intrapersonal and behavioral dimensions of LA, empowering students to take initiative and take responsibility for their learning journeys.

Hernandez et al. reported that students exhibit good self-regulation and excellent reflection but show only competence in planning, monitoring, and control [54]. This has been explained by the presence of the Kruger–Dunning effect, where students overestimate their abilities, underscoring the importance of accurate self-assessment [71]. Studies have also revealed that self-assessment training improves SRL abilities such as goal setting, attention focusing, and self-reflection. Positive attitudes toward self-direct observation of procedural skills and improvements in clinical performance highlight the role of self-assessment in fostering SRL and learner agency, as students become more adept at monitoring and directing their learning [59, 60]. Additionally, a transition from a deep learning approach to a surface learning approach was noted in a study as students moved to clinical training [50]. These insights collectively underscore the pivotal role of behavioral strategies in enhancing students’ cognitive engagement and fostering adaptive learning practices within academic contexts.

Clinical reasoning skills are critical for developing students’ ability to analyze and synthesize information in clinical contexts, thereby fostering deeper engagement and motivation [64]. Similarly, the clinical application of skills reinforces motivation by showcasing the direct impact of learning on patient care and outcomes, thereby enhancing students’ sense of professional efficacy and fulfillment in their studies [57, 59]. The transition from preclinical to clinical courses has shown a shift from controlled to autonomous motivation [58]. These processes align with LA principles by enabling students to take ownership of their professional growth through hands-on experience and critical thinking.

Among all the articles included in this review, only one addressed learner agency in undergraduate dental education, and it explored diverse student perspectives encompassing career readiness, the efficacy of PBL, reliance on faculty support, and the cultivation of professional identity. PBL is instrumental in fostering learner agency by promoting autonomy, problem-solving skills, and interpersonal competencies among students [5]. While PBL enhances these crucial skills, it may also present challenges such as increased workload [61]. The quantitative findings also underscore the detrimental effects of increased workload on students during their clinical transition. Initially, this heightened workload is associated with negative impacts; however, over time, students tend to cultivate resilience, heightened motivation, and a more profound sense of professional identity [66].

The current study has several limitations that need to be acknowledged. First, the search strategy included only key databases, which means that some relevant articles might have been overlooked. Moreover, the inclusion criteria may have introduced bias, potentially leading to the exclusion of some articles. This limitation could result in an incomplete representation of the literature. Second, the review was restricted to articles published in the English language and focused specifically on undergraduate dental education. This language- and field-specific focus may have excluded valuable insights from non-English publications and other educational disciplines. Furthermore, none of the reviewed articles explicitly conceptualized LA; instead, they used the terms associated with LA as common concepts without a clear framework. This lack of explicit conceptualization highlights a gap in the literature that future research should address. Importantly, this article is a scoping review, not a systematic review. As such, we did not critically appraise the selected studies, which is a common practice in systematic reviews, to assess the quality and reliability of the evidence. However, despite this limitation, we successfully located findings that addressed our aim of examining the nature and extent of the literature on LA, SRL, and SDL and identifying potential future research directions. It must be reiterated that student empowerment as independent learners must be balanced with appropriate support and mentoring by faculty for a balanced and comprehensive professional development of students. These findings provide a foundational understanding but also underscore the need for more rigorous and comprehensive studies to advance the field further. Additionally, robust assessment tools should be developed, and collaboration among dental educators should be promoted to share best practices such as flipped classrooms, blended learning, case-based learning etc. while including emerging technologies such as artificial intelligence [21, 56, 65, 72, 73]. To build on these findings, future steps should include empirical validation of the proposed framework linking LA, SRL, and SDL in dental education.

## Conclusion

This study provides insights into how SRL and SDL foster student agency, particularly in areas such as self-efficacy, motivation, goal setting and peer support in undergraduate dental education. SRL and SDL may contribute to the professional development of undergraduate students into independent learners beyond the temporal confines of the university environment, play a potentially useful role in their transition into clinical practice and facilitate the acquisition of lifelong learning behaviors. Institutions might consider developing strategies that encourage the transformation of students into independent learners with less reliance on faculty for their academic progress. Learner independence requires a greater focus than information transfer from teachers to students. Educational strategies that facilitate LA development may facilitate the transition of students into clinical practice. Future research should follow a theory-driven and evidence-based approach to design educational activities to support students to develop their agency through diverse activities.

## Electronic supplementary material

Below is the link to the electronic supplementary material.

Supplementary Material 1

Supplementary Material 2

## Data Availability

No datasets were generated or analysed during the current study.

## Abbreviations

LA: Learner Agency
SRL: Self-Regulated Learning
SDL: Self-Directed Learning
HPE: Health Professions Education
IRR: Interrater Reliability
PBL: Problem-Based Learning
