# Supplementary material for: Beyond autonomy: unpacking self-regulated and self-directed learning through the lens of learner agency- a scoping review
Source: BMC Med Educ. 2024 Dec 23;24:1519. doi: 10.1186/s12909-024-06476-x (PMC11667877; doi:10.1186/s12909-024-06476-x)
Supplement: Supplementary file 1 — Supplementary Material 1 [file 12909_2024_6476_MOESM1_ESM.docx]

| Stages | Description | Page |
| --- | --- | --- |
| 1. Identifying the research question | The research question was identified to cover a wide range of literature | 8 |
| 1. Identifying relevant studies | A systematic search of electronic databases and the reference lists of key papers was conducted using keywords pertaining to self- regulated learning, self-directed learning and learner agency in undergraduate dental education. | 8-10 |
| 1. Study selection | Papers were screened for inclusion using the inclusion and exclusion criteria. The literature search and study selection are depicted in a flowchart. A codebook encompassing learner agency themes and references was established, and participant information was extracted. | 10-12 |
| 1. Charting the data | Data extracted from the papers included the author’s name, year of publication, source, type, country, research method, theories and analytical framework, participants, context, and learner agency themes. | 12-15 |
| 1. Collating, summarizing and reporting results | Findings were summarized and reported using thematic analysis to interpret the results  and provide context for the research. | 15-19 |

**Arksey and O’Malley Framework Checklist**
